# Tetramic and Tetronic Acids as Scaffolds in Bioinorganic and Bioorganic Chemistry

**DOI:** 10.1155/2010/315056

**Published:** 2010-05-25

**Authors:** G. Athanasellis, O. Igglessi-Markopoulou, J. Markopoulos

**Affiliations:** ^1^Laboratory of Organic Chemistry, School of Chemical Engineering, National Technical University of Athens, 15773 Athens, Greece; ^2^Laboratory of Inorganic Chemistry, Department of Chemistry, University of Athens, Panepistimiopolis, 15771 Athens, Greece; ^3^ALAPIS Pharmaceuticals, R & D Centre, Pallini, 15302 Attiki, Greece

## Abstract

Tetramic and tetronic acids are naturally occurring molecules with a variety of biological activities. In this review article, we present the general strategies for the synthesis of these compounds and we reveal the functionalized groups that are responsible for their properties. We also set out their coordinating modes with up-to-date bibliographical references.

## 1. Introduction

Tetramic acids, pyrrolidine-2,4-dione derivatives, are naturally occurring molecules synthesized by numerous organisms and found in a variety of natural products [1, 2]. This class of five membered heterocycles has attracted significant attention due to the broad range of biological activities they exhibit. This activity comprises of antibiotic and antiviral, cytotoxicity, mycotoxicity, as well as inhibition of the cell cycle. Various examples of tetramic acid derivatives isolated from the nature are streptolydigin which inhibits RNA polymerase [3], the melophlin family of compounds which have shown antimicrobial activity [4], equisetin and its homologue trichosetin with inhibitory activity against Gram positive bacteria [5, 6], and reutericyclin which exhibits a wide range of pharmacological activities [7, 8]. In addition, a series of derivatives have been patented by Bayer CropScience as ingredients for fungicidal and herbicidal use [9]. 

On the other hand, tetronic acids, 4-hydroxy-[5H] furan-2-ones, are compounds with antibiotic, antiviral, antineoplastic, and anticoagulant activity [10, 11]. Compounds which have been isolated from natural products and exhibit such activity are tetronasin [12], RK-682 [2, 13], the well-known family of compounds named vulpinic acids [14, 15] and many others.

For a long time, we have been involved in the chemistry of tetramic and tetronic acids and the design of new strategies for the preparation of small heterocyclic molecules. Their synthesis has been accomplished based on a similar strategy starting from the appropriate precursors, suitably protected *α*-amino acids for tetramic and *α*-hydroxy acids for tetronic acids, using the N-hydroxybenzotriazole methodology for the synthesis of their active esters.

## 2. Synthesis of Tetramic Acids

**Scheme 1:**
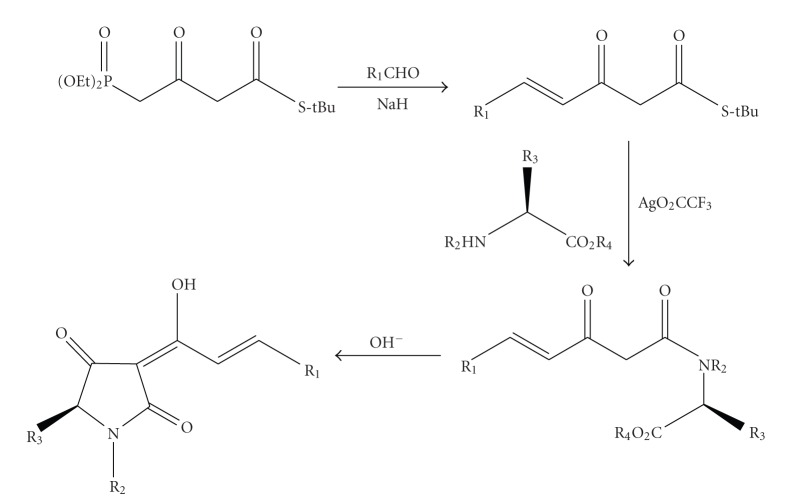
Synthesis of optically active tetramic acids by Leyet al. [16].

Owing to the importance of tetramic acid derivatives, numerous approaches to their synthesis have been developed. They mainly make use of amino acid-derived precursors whose stereochemical integrity remains more or less conserved in the structure of the products. Significant studies on the synthesis of such optically active compounds have been made by Ley et al. [16] who used a series of *β*-ketoamides as intermediates for the preparation of enantiomerically pure 3-acyl tetramic acids, based on the Lacey methodology for the synthesis of tetramic acids by N-acylation of *α*-amino acids (Scheme 1). 

**Scheme 2:**
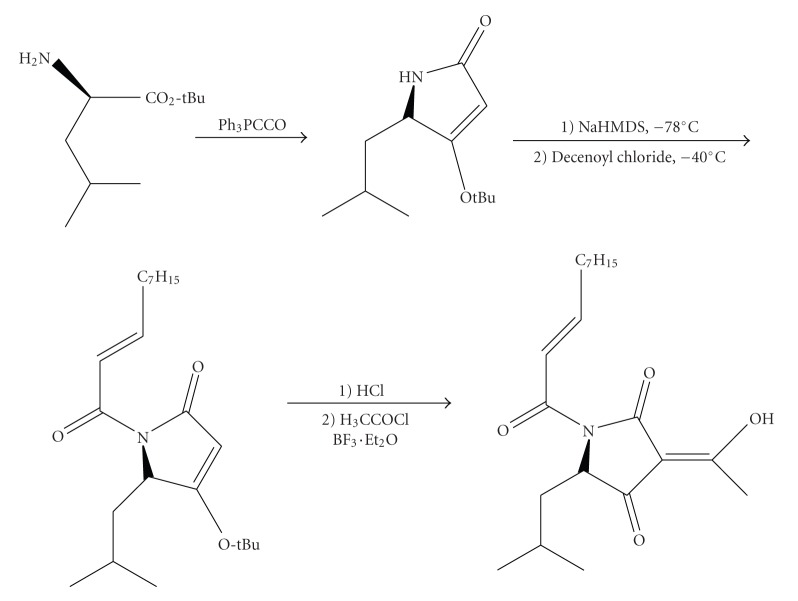
Synthesis of reutericyclin by Schobertand Jagush [21].

On the other hand,Andrews et al.[17] provided an N-acyloxazolidine derivative of L-serine as a suitable precursor for the construction of chiral substituted tetramic acids with high enantiomeric excess. Other methodologies based on the enantioselective Lacey-Dieckmann cyclization, requiring strongly basic conditions, have also been reported [18, 19] whereas Jouin and coworkers have proposed the use of Meldrum acid in the presence of isoprenyl chloroformate and DMAP reagents [20]. Recently,Schobert and Jaguschproposed an expedient synthesis of tetramic acids from *α*-amino esters, in which the cyclization route involved a domino addition-Wittig alkenation reaction with immobilized triphenylphosphoranylidene ketene under neutral nonracemizing conditions [21]. Acylation to 3-acyltetramic acids was then performed with the appropriate acyl chloride and boron trifluoride-diethyl etherate under microwave irradiation. This route was followed in the synthesis of natural products like reutericyclin (Scheme 2).

**Scheme 3:**
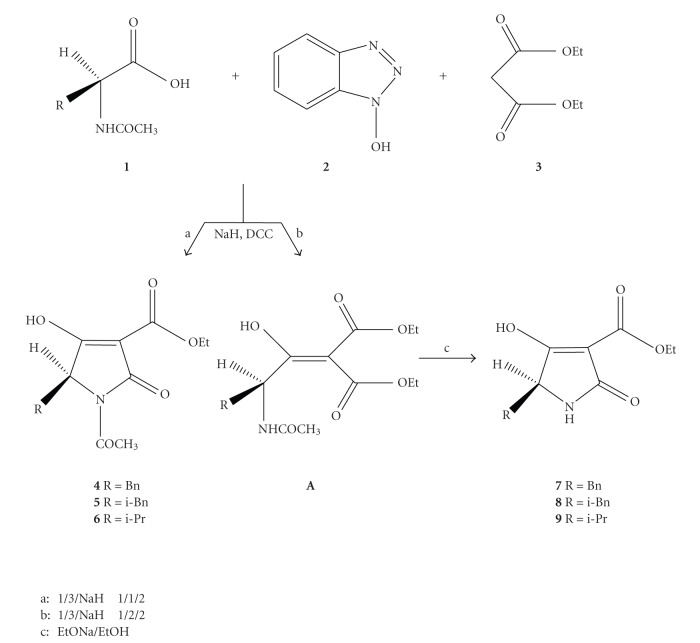
Synthesis of tetramic acids.

Our first attempt to use N-hydroxybenzotriazole in the synthesis of heterocyclic compounds was made in the field of tetramic acids [22]. We applied the "one-pot" synthetic strategy which comprises of a C-acylation reaction between the N-hydroxybenzotriazole ester of the appropriate optically active amino acid **1** and diethyl malonate **3**. When the product was not the corresponding tetramic acid **4**–**6** but the C-acylation compound **A**, a cyclization reaction under basic conditions was performed to afford the corresponding tetramic acid **7**–**9** (Scheme 3).

The crucial parameter on the synthesis of the N-acylated-3-ethoxycarbonyl tetramic acids **4**–**6** or N-H-3-ethoxycarbonyl tetramic acids **7**–**9** is the molar ratio between the N-acylated amino acid **1** and diethyl malonate **3**. We observed that when diethyl malonate **3** was used in molar excess (2 equiv.), the oily product containing the C-acylation compound **A** and diethyl malonate **3** was obtained. On the other hand, when diethyl malonate **3** was used in stoichiometric ratio (1 equiv.), the N-acetyl-3-ethoxycarbonyl tetramic acids **4**–**6** were obtained as white solids. The enantiomeric purity of the final products was tested by HPLC and the results were in the range 82**%–**96%ee.

These results indicate the success of the proposed methodology to maintain the stereochemical integrity of the corresponding *α*-amino acids. Another advantage of the proposed methodology is that there is no need for isolating the intermediate N-hydroxybenzotriazole esters of the chiral *α*-amino acids, in contrast to previously described methodologies. This fact reduces the time for the synthesis of the desired products and is beneficial for the overall yield of the reaction (45%–75%). Therefore, the reaction is simple, inexpensive, easily scaled up and proceeds with low racemization.

## 3. Synthesis of Tetronic Acids

Given our interest on the synthesis of tetramic acids and their coordination compounds, we have oriented our interest on the chemistry of tetronic acids.

**Scheme 4:**
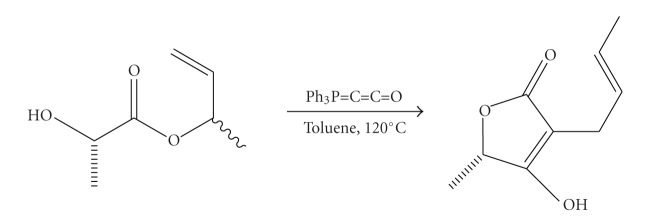
Synthesis of tetronic acids bySchobert et al. [30].

In the literature, there are a number of reliable methods for the synthesis of such derivatives. Several methodologies include Dieckmann cyclization [23], cycloaddition [24], oxidation [25], Wittig-Claisen [26], lactonization [27], and enzymatic reactions [28]. A few years ago, the synthesis of 3-acyl-5-methoxycarbonyl tetronic acids has been reported from our research group [29]. A new strategy for the synthesis of functionalized tetronic acids was developed by Schobert and coworkers [30], applying the “domino” process, which comprises of the reaction between the esters of *α*-hydroxy acids and the cumulated phosphorus ylide ketenylidenetriphenylphosphorane (Scheme 4). 

**Scheme 5:**
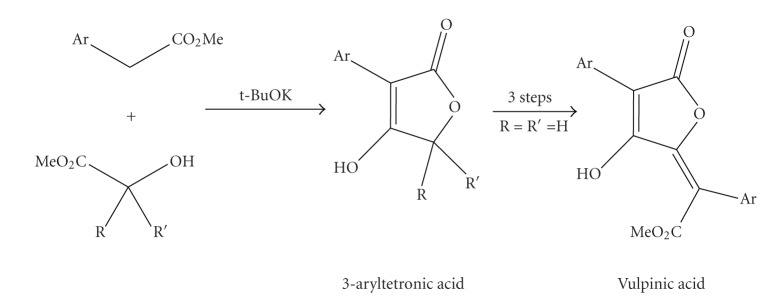
Synthesis of 3-aryltetronic acids as precursors for vulpinic acids byMallinger et al. [31].

In addition, the one pot synthesis of 3-aryl- unsubstituted, mono- and disubstituted at 5 position of the heterocyclic nucleus tetronic acids, as well as three natural vulpinic acids have been recently studied byMallignerand coworkers based on a tandem transesterification/Dieckmann condensation reaction (Scheme 5) [31].

**Scheme 6:**
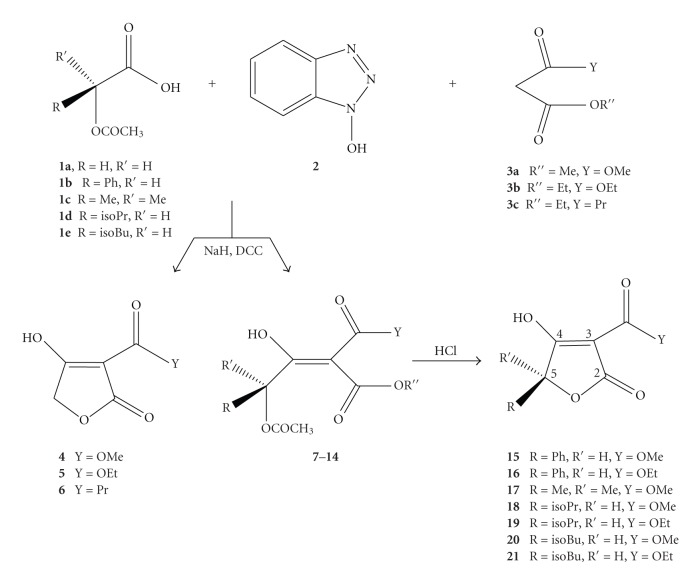
Synthesis of tetronic acids.

As a logical extension of our previous efforts on the synthesis of small heterocyclic compounds, we decided to investigate the condensation reaction of N-hydroxybenzotriazole esters of O-protected *α*-hydroxy acids and active methylene compounds bearing appropriate substituents suitable for preparing highly functionalized tetronic acid derivatives with pharmacological interest [32]. Generally, a definite short-step methodology for producing chiral 3-substituted tetronic acids or their *γ*-hydroxy ester precursors via a C-acylation reaction between the N-hydroxybenzotriazole ester of an appropriate O-protected-*α*-hydroxy acid and the desired active methylene compound was accomplished (Scheme 6).

The proposed strategy comprises of a C-acylation reaction between an active methylene compound **3** and the N-hydroxybenzotriazole ester of the appropriate O-protected-*α*-hydroxy acid **1**. In cases where the main product of the C-acylation reactions were the functionalized 4-acetoxy-3-hydroxybutenoates **7**–**14**, we used these *γ*-hydroxy esters for the preparation of the corresponding tetronic acid derivatives **15**–**21** under acidic conditions (MeOH, 10% HCl). At this point, it is important to notice that the lactonization of the *β*-hydroxybutenoates proceeded without racemization of stereogenic centers at C-5. One first remark in our proposed synthetic route is that only the O-acetyl-glycolic acid **1a** gave the corresponding tetronic acids **4**–**6** via one-step reaction. In contrast, S-mandelic acid **1b**, *α*-hydroxyisobutyric acid **1c**, L-*α*-hydroxyisovaleric acid **1d**, and L-*α*-hydroxyisocaproic acid **1e** gave the corresponding *γ*-acetoxy-*β*-hydroxybutenoates **7**–**14** as oily products. These intermediates were treated with 10% HCl in MeOH at room temperature for 24 or 48 hours to afford the corresponding tetronic acids **15**–**21**. Additionaly, the tetronic acids **15**, **16**, and **18**–**21** have been found to be optically active as shown by their optical rotations. This observation is in full accordance with the results obtained in the synthesis of tetramic acids [22].

## 4. Tetramic Acids as Quorum Sensing Molecules

The survival of microorganisms may contain the mechanism of eliminating the presence of other such organisms through the destruction of their transmembrane permeability. In such a context, the scientific team of D. K. Janda has extensively studied the role of 3-oxo-dodecanoyl homoserine lactone (3-oxo-C_12_-HSL) in *P. aeruginosa* sp [33–35]. Based on the fact that 3-oxo-C_12_-HSL can be easily converted to the corresponding tetramic acid (C_12_-TA) through a nonenzymatic Claisen “internal rearrangement”, the antimicrobial activity of these two compounds was examined. Therefore, 3-oxo-C_12_-HSL has an action mainly on host cells acting as “quorum sensing” molecule (QS), whereas its conversion to the C_12_-TA is important in order to inhibit the life of bacterial competitors. In addition, C_12_-TA has no inhibitory activity on mammalian cells in contrast to other tetramic acids which were developed as potent antibiotics [36, 37] and the precursor 3-oxo-C_12_-HSL. Although the mode of action of tetramic acid remains to be established, this molecule is a potent “iron chelator” (see [35] and references therein). Consequently, it is clear that the discovery and synthesis of tetramic acids which are derived from naturally existing “homoserine lactones” is a new challenging avenue in medicinal chemistry.

## 5. Tetramic Acid Coordination Compounds

Early studies on fungal toxins have demonstrated that tetramic acids tend to occur naturally as metal-chelate complexes [38]. Metal chelation by tetramic acid nucleus seems to be important for transport across membranes in biological tissues [2]. Tetramic acids possessing a 3-acyl group have the ability to chelate divalent metal ions. For instance, tenuazonic acid from the fungus *Phoma sorghina* has shown to form complexes with Ca(II) and Mg(II) [39] as well as heavier metals such as Cu(II), Ni(II), and Fe(III) [40, 41]. Furthermore, the research group ofBiersack et al.has extensively studied melophlins, a group of 3-acyl-N-methyl tetramic acids, as far as their synthesis andbiological activity is concerned,and ithas presented the synthesis of complexes of melophlins with Mg, Zn, Ga, La, and Ru [42]. The chelation mode is the well-known complexation through the oxygen atom of the exocyclic carbonyl group (attached at position 3 of the heterocyclic nucleus) and the ketonic moiety of position 4 (E-isomer) or position 2 (Z isomer), respectively (Scheme 9). The biological evaluation of the new complexes showed antiproliferative activity against various cancer cells. Likewise, cyclopiazonic acid (CPA) [43] is a toxic indole tetramic acid produced by various fungi and found to inhibit SERCA (a well-studied member of the P-type ATPase family in the rabbit skeletal muscle). The way CPA works was studied through its chelation mode with Mg(II), Mn(II), and Ca(II), and it was revealed that the bivalent way of chelation is desirable in order to enhance the cytoplasmic cation access pathway. 

Our research group interest deals with the coordination capabilities of various heterocyclic compounds containing the *β*,*β*′-dicarbonyl system. Among others, we have prepared new metal complexes with pyrrolidine-2,4-dione derivatives, in order to improve their pharmacological profile by binding them to metal ions. As it was already reported in the literature [1, 2], the biological activity of some tetramic acid derivatives significantly has been enhanced by binding to metal ions. It was found that in some cases the metal complexes obtained revealed higher biological activity than their ligands.

**Scheme 7:**
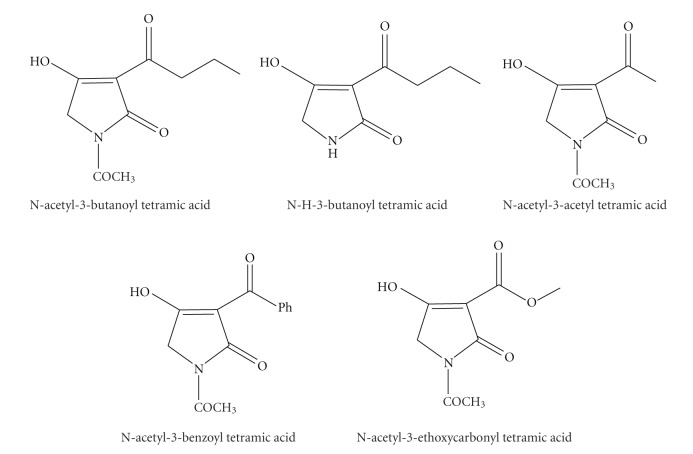
3-substituted tetramic acids.

Over the past years, we synthesized two novel ligands based on tetramic acid core, the N-acetyl-3-benzoyl and 3-butanoyltetramic acids, with binding sites suitable for chelation of Co(II), Ni(II), Cu(II), Cd(II), and Hg(II) species [44] (Scheme 7). Starting from these ligands, complexes with 1 : 1 and 1 : 2 metal to ligand stoichiometries were prepared. The magnetic and spectroscopic properties of the Co(II), Ni(II), and Cu(II) halide and thiocyanate complexes of formula MX_2_L (L = tetramic acid ligand) indicate that these contain six-coordinated metals with both bridging anions and tetramic acids. The acids appear to be bonded to the metals possibly through the nitrogen atom and a carbonyl oxygen atom and the IR spectra indicate the Cd(II) and Hg(II) complexes to be possibly tetrahedral.

The rhodium (I) complexes [Rh(acac){P(OPh)_3_}_2_] and [Rh(acac)(CO)PPh_3_] (acac = acetylacetonate) in the presence of triphenyl-phosphite or phosphine, respectively, are catalyst precursors for the hydroformulation of olefins under mild conditions [45]. The substitution of acac by other chelating molecules, including the *β*-diketonate, moiety has been less well studied for rhodium complexes.

The preparation of rhodium(I) complexes containing the N-acetyl-3-butanoyltetramic acid (Habta) together with their structural characterization via X-ray analysis of [Rh(abta){P(OPh)_3_}_2_], their ^1^H, ^13^C, ^31^P NMR spectra, and IR measurements have been investigated [46].

The addition of 1 equiv of Habta to a solution of [Rh(acac)(CO)_2_] in CH_2_Cl_2_ results in complete substitution of acac by abta with formation of [Rh(abta)(CO)_2_] which underwent displacement of CO by either P(OPh)_3_ or PPh_3_ to give [Rh(abta)(CO)L] L = P(OPh)_3_ or PPh_3_ and [Rh(abta){P(OPh)_3_}_2_]. 

The ^13^C NMR spectrum of [Rh(abta)(CO)_2_] consists of two equally intense resonances due to rhodium carbonyls which are equivalents as a result of the asymmetry of coordinated abta. Spectroscopic data for all the abta complexes are consistent with its coordination O,O′-mode through the functionalities associated with C(4) and the acyl group at C(3) in the pyrrolidinone ring, as by X-ray crystallography. The IR spectrum of [Rh(abta)(CO)_2_] showed two equally intense *v*(CO) bands at 2095 and 2027 cm^−1^ owing to the fast substitution of acac (*v*CO 2085, 2014 cm^−1^ in [Rh(acac)(CO)_2_]), whereas a strong absorption in the range 1605–1612 cm^−1^ can be attributed to a combination of the *v*(CO) and *v*(C=C) vibrations of coordinated data.

**Scheme 8:**
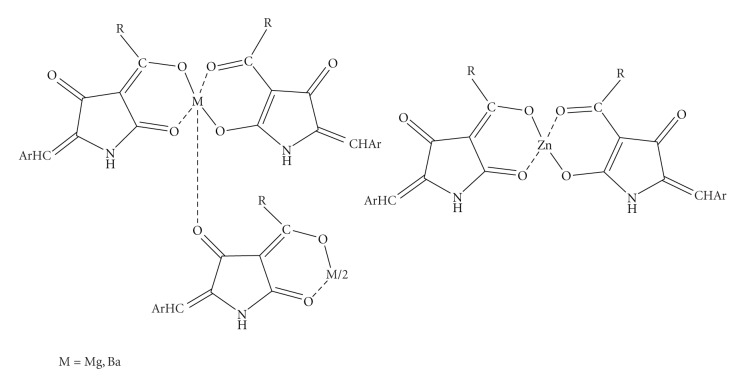
Metal complexes of 5-benzylidene-3-acyl tetramic acids.

The 5-arylidene-3-alkanoyl tetramic acids contain important structural adjuncts, namely, an enolic *β*,*β*′-tricarbonyl moiety, a lipophilic 3-alkanoyl substituent, and a hydrophobic group at the 5-position which allow them to anticipate versatile activity. Moreover, the *β*, *β*′-tricarbonyl moiety provides them with sites available for metal complexation. These properties prompted us to study the synthesis and the complexation reaction of 5-benzylidene-3-hexanoyl tetramic acid (BHTA) with the halides of Mg(II), Ba(II), and Zn(II) [47]. Interest in complexes of Mg(II) arises from the antibiotic “Magnesidin”, containing the 5-ethylidene-3-alkanoyl acids with Mg(II) [48]. The structure of the novel complexes of Mg(II) and Ba(II) followed the pattern of two metal ions and three ligands in the complex structure whereas the complexation reaction with Zn(II) halide afforded a complex comprising of the metal ion and two ligands (Scheme 8). Elemental analyses and FAB MS spectra revealed structures of the formulae Mg_2_L_3_(OH)·4H_2_O, Ba_2_L_3_(OH)^.^6H_2_O, and ZnL_2_·4.5H_2_O. In the ^13^C NMR spectra of the complex with Zn, the appearance of two signals at different values for each carbonyl carbon is the proof of the existence of two five-membered inequivalent chelate rings, whereas in the complexes of Mg and Ba the NMR spectra exhibit three resonances for the carbonyl carbons. These signals are not equally intense, an indication for the presence of three tautomers which are interconverted by a relatively slow metal-oxygen dissociation-association process on the NMR time scale. 

The structural investigation of the metal 5-benzyliden-3-alkanoyl tetramic acid is important to analyze both the ligating abilities of tetramic acids and the effects of coordination on the conformation of the HL/L^−^ molecules.

**Scheme 9:**
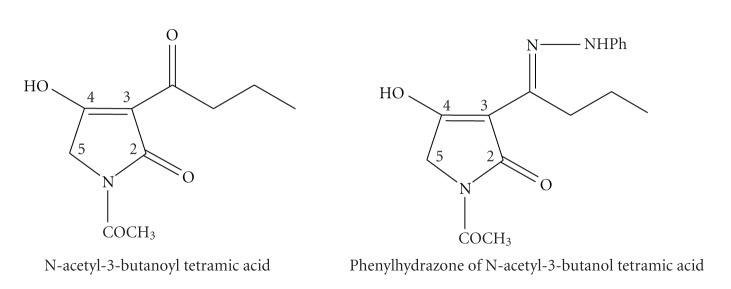
N-acetyl-3-butanoyl tetramic acid and its phenylhydrazone derivative.

It is well-known that N and O play a key role in the coordination of metals at the active sites of numerous metallo-biomolecules. Therefore, a number of Cu(II), Co(II), Ni(II) and Zn(II) acetate complexes containing the enolate N-acetyl-3-butanoyltetramic acid and its phenylhydrazone derivative analogues were studied [49]. The reaction in 1 : 1 ratio afforded complexes of the general formula M(OAc)(L-H)·H_2_O whereas the reaction in 1 : 2 ligand to metal ratio gave complexes of the formula M(L-H)_2_·*y*H_2_O. The way the ligand is complexed to the metal ion was proved by X-ray analysis of the crystals obtained from the reaction of the ligand with Cu(OAc)_2_·H_2_O. The enolate of the ligand is complexed through the oxygen atoms of the hydroxyl group of position 4 and the carbonyl oxygen of the acyl moiety attached at position 3 of the heterocyclic ring. In this complex, copper possibly adopts a slightly distorded octahedral coordination geometry. The reaction of the ligand with Zn(II) acetate in 1 : 1 and 1 : 2 ratio, respectively, gave complexes where the depronotaded ligand was further deacylated at the nitrogen atom in the first situation but not in the second one. In addition, a new ligand was then synthesized, the phenylhydrazone of the previously used tetramic acid (Scheme 9), and its complexes with Cu(II) and Co(II) in 1 : 1 and 1 : 2 ratio were formed. The structures exhibited the general formulae M(OAc)(L-H) and M(L-H)_2_, respectively, as described for the tetramic acid. In contrast to the situation with Zn(II) acetate, the reaction of the phenylhydrazone of tetramic acid with Zn(OAc)_2_·2H_2_O irrespective of the metal to ligand ratio afforded Zn(OAc)(L-H) containing the deacylated ligand. 

Finally, the solid state structure of [Cu(abta)_2_ (py)_2_]·2H_2_O has been determined by single crystal X-ray diffraction. It shows that copper adapts a slightly distorted octahedral coordination geometry with ligand adopting an O,O′-mode of coordination via the functionalities associated with C4 and the acetyl group at C3 in the pyrrolidine ring.

**Scheme 10:**
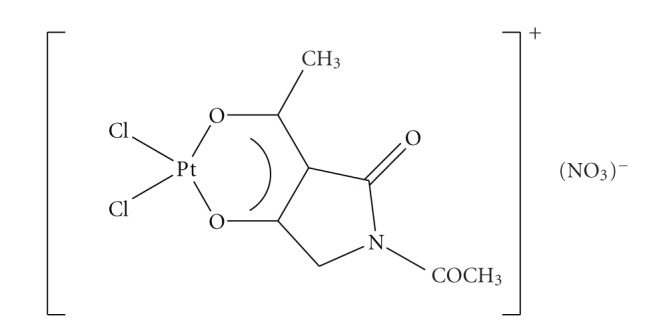
The cationic diamineplatinum tetramic acid complexes.

New platinum (II) complexes containing 3-alkanoyl tetramic acids have shown to exhibit a broad spectrum of biological properties. Although the synthesis and the antitumor activity of these complexes is mentioned in two patents [50, 51], no details are given concerning the structure of the complexes. There is a large body of experimental evidence suggesting that the success of platinum complexes in killing tumor cells results from the ability to damage DNA by forming various types of covalent adducts [52, 53]. Encouraged by promising chemotherapeutic properties of “cisplatin” complexes, we investigated the coordination ability of N,3-diacetyl tetramic acid (Hata) with *cis*-(NH_3_)_2_PtCl_2_, (dach)PtCl_2_, (en)PtCl_2_^,^ and K_2_PtCl_4_. The structure of the isolated complexes was investigated by means of IR, NMR, ESI-MS Spectroscopy, and molar conductivity measurements [54].The pattern of complexation of the deprotonated ligand follows possibly the known bidentate mode through the oxygen atoms of the 3-acyl moiety and the hydroxyl group of position 4 of the heterocyclic nucleus (Scheme 10). The coordination sphere around Pt(II) can be described as distorted square-planar and the stability of the ligand in its complexation ability remains except for the situation of performing the NMR experiments in DMSO-d_6_ in which the ligand is fully replaced by the solvent molecules in the metal complex. These complexes have similarities with complexes between alkanoyl tetramic acids and Pt(II) which were patented since they exhibited interesting biological activities. In this context the structure evaluation of our complexes is very useful in order to perform structure-activity relationship experiments with the previous complexes. 

**Scheme 11:**
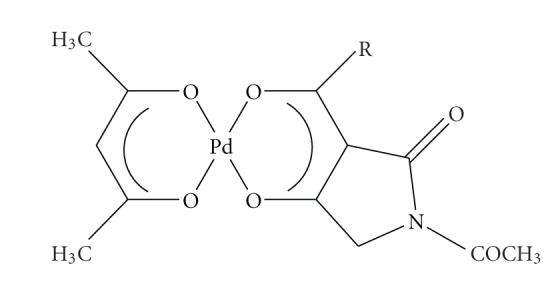
Pd complexes of substituted tetramic acids.

The ability of N-acetyl-3-butanoyltetranic acid (Habta)enolate ligand to substitute acetylacetonate from [Rh(acac)(CO)_2_] prompted us to study the progressive displacement of acac from [Pd(acac)_2_] complexes which occurs on reaction with different tetramic acids [L = N,3-diacetyl (Hata), N-acetyl-3-butanoyl (Habta), and N-acetyl-3-ethoxycarbonyl (Haceta)] [55]. In the first two situations (3-acyl and 3-butanoyl tetramic acids), the displacement afforded complexes of the general formulae [Pd(acac)(L-H)] in 1 : 1 and 1 : 2 ratio of reaction, but in the situation of 3-ethoxycarbonyl tetramic acid the only isolated complex was [Pd(acac)(L-H)] even in 1 : 4 ratio of reaction. On the other hand, the reaction of all the above tetramic acids in aqueous solution of K_2_[PdCl_4_] gave complexes of the general formula [Pd(L-H)_2_]. The study of the structure of complexes with NMR Spectroscopy showed that there is only one isomer in complexes [Pd(acac)(L-H)] whereas in complexes [Pd(L-H)_2_] two isomers are apparent, which are evaluated as the “*cis*” and “*trans*” isomers based on the possible bidentate complexation of the ligand through the oxygen atoms of the pyrrolidine nucleus (Scheme 11). Addition of a Lewis base, such as pyridine, to a chloroform solution of [Pd(abta)_2_], forms a Lewis base adduct, [Pd(py)_4_(abta)_2_] which has been characterized by X-ray analysis and shown to contain a square planar Pd(py)_4_ group with *trans*-monodentate weeky bonded abta groups. 

## 6. Tetronic Acid Coordination Compounds

The coordination mode of tetronic acids is a research field with great interest and many examples in the recent literature can be found. Complexes of tetronic acids with Cu(II) have been synthesized and their biological activity was elucidated [56], whereas a number of complexes of 3-acyl tetronic acids with Pd(II) and Pt(II) have also been reported [57, 58]. Finally, complexes with several metal ions have proved the existence of 1 : 2 or 1 : 3 ratios (metal : ligand) either by conductometric or pH-metric titrations [59] or by X-Ray crystallographic analysis [60].

**Scheme 12:**
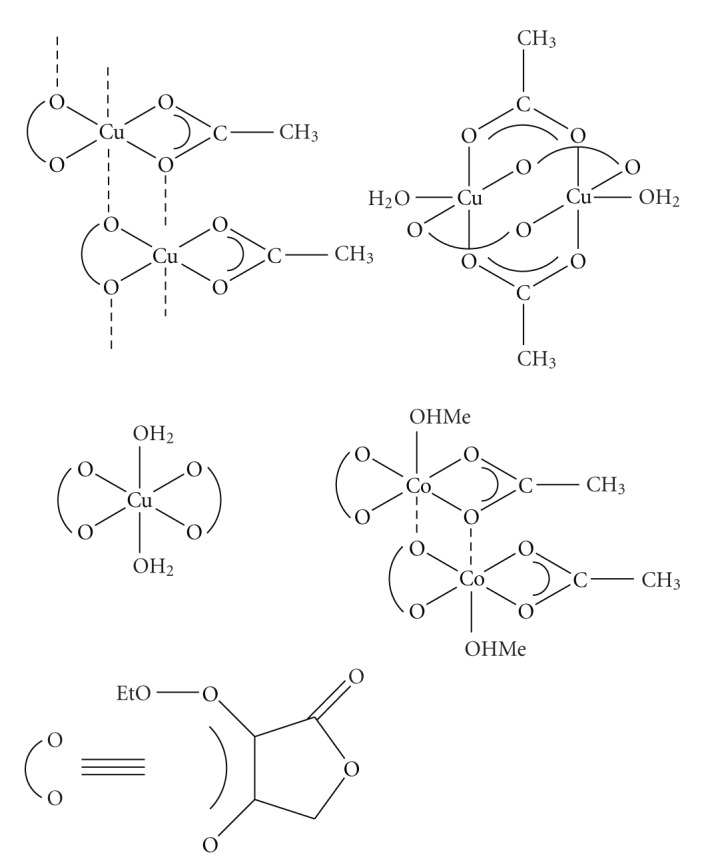
Metal complexes of substituted tetronic acids.

The complexation mode of 3-ethoxycarbonyl tetronic acid (L = HETA) with acetates and chlorides of Cu(II) and Co(II) was studied based on measurements of magnetic susceptibility and EPR Spectroscopy [61]. The complexes isolated were Cu(OAc)(L-H), Cu_2_(OAc)_2_(L-H)_2_(H_2_O)_2_, Cu(L-H)_2_(H_2_O)_2_^,^ and Co_2_(OAc)_2_(L-H)_2_(MeOH)_2_ (Scheme 12). The isolated complexes of Cu(II) and Co(II) acetates with HETA in 1 : 1 ratio have a possible octahedral stereochemistry with bidentate coordination mode through O(4) and O(6) of the tetronate ring as indicated by the shift to lower wavenumbers of the lactone and diketone characteristic bands. In addition, magnetic susceptibility measurements showed that no reduction to Cu(I) occurred whereas the result for Co(II) complex gives evidence of octahedral stereochemistry. The chloride Cu(II) complex with HETA in 1 : 2 ratio has a possible octahedral stereochemistry, whereas the exclusion of dinuclear species was achieved through EPR measurements. However both Cu(II) compexes showed the presence of two sets of EPR signals indicating an unhomogenity of centers; some of them point to a mononuclear structure, while the others adopt a dinuclear structure. Moreover, EPR studies for these compounds showed the possible mononuclear and dinuclear structures, respectively. In summary, we have prepared a plausible model for the copper, cobalt *β*, *β*′-tricarbonyl coordination compounds. Our proposed model may help define some of the unusual features associated with copper and cobalt metallobiochemistry.

## References

[1] Royles BJL (1995). Naturally occurring tetramic acids: structure, isolation, and synthesis.

[2] Schobert R, Schlenk A (2008). Tetramic and tetronic acids: an update on new derivatives and biological aspects.

[3] Tuske S, Sarafianos SG, Wang X (2005). Inhibition of bacterial RNA polymerase by streptolydigin: stabilization of a straight-bridge-helix active-center conformation.

[4] Aoki S, Higuchi K, Ye Y, Satari R, Kobayashi M (2000). Melophlins A and B, novel tetramic acids reversing the phenotype of ras- transformed cells, from the marine sponge Melophlus sarassinorum.

[5] Phillips NJ, Goodwin JT, Fraiman A, Cole RJ, Lynn DG (1989). Characterization of the Fusarium toxin equisetin: the use of phenylboronates in structure assignment.

[6] Marfori EC, Kajiyama S, Fukusaki E-I, Kobayashi A (2002). Trichosetin, a novel tetramic acid antibiotic produced in dual culture of Trichoderma harzianum and Catharanthus roseus callus.

[7] Holtzel A, Ganzle MG, Nicholson GJ, Hammes WP, Jung G (2000). The first low molecular weight antibiotic from lactic acid bacteria: reutericyclin, a new tetramic acid.

[8] Marquardt U, Schmid D, Jung G (2000). Racemic synthesis of the new antibiotic tetramic acid reutericyclin.

[9] Fischer R, Lehr S, Feucht D 2-ethyl-4,6-dimethyl-phenyl-substituted tetramic acid derivative
as pest control agents and/Or herbicides.

[10] Tejedor D, García-Tellado F (2004). Synthesis and chemistry of tetronic acids.

[11] Zografos AL, Georgiadis D (2006). Synthetic strategies towards naturally occurring tetronic acids.

[12] Davies DH, Snape EW, Suter PJ, King TJ, Falshaw CP (1981). Structure of antibiotic M139603; x-ray crystal structure of the 4-bromo-3,5-dinitrobenzoyl derivative.

[13] Roggo BE, Petersen F, Delmendo R, Jenny HB, Peter HH, Roesel J (1994). 3-alkanoyl-5-hydroxymethyl tetronic acid homologues and resistomycin: new inhibitors of HIV-1 protease I. Fermentation, isolation and biological activity.

[14] Pattenden G, Turvill MW, Chorlton AP (1991). Maleic anhydrides in synthesis. Preparation of furan-2(5H)-one phosphonate derivatives and a new synthesis of pulvinic acids and pulvinone analogues.

[15] Bourdreux Y, Bodio E, Willis C, Billaud C, Le Gall T, Mioskowski C (2008). Synthesis of vulpinic and pulvinic acids from tetronic acid.

[16] Ley SV, Smith SC, Woodward PR (1992). Further reactions of t-butyl 3-oxobutanthioate and t-butyl 4-diethyl-phosphono-3-oxobutanthioate: carbonyl coupling reactions, amination, use in the preparation of 3-acyltetramic acids and application to the total synthesis of fuligorubin A.

[17] Andrews MD, Brewster AG, Crapnell KM (1998). Regioselective Dieckmann cyclisations leading to enantiopure highly functionalised tetramic acid derivatives.

[18] Paquette LA, Macdonald D, Anderson LG, Wright J (1989). A triply convergent enantioselective total synthesis of (+)-ikarugamycin.

[19] Jones RCF, Tankard M (1991). A new sequence for the synthesis of 3-(poly)enoyltetramic acids.

[20] Jouin P, Castro B, Nisato D (1987). Stereospecific synthesis of N-protected statine and its analogues via chiral tetramic acid.

[21] Schobert R, Jagusch C (2005). An expedient synthesis of 3-acyltetramic acids of the melophlin family from *α*-aminoesters and immobilized Ph_3_PCCO.

[22] Athanasellis G, Gavrielatos E, Igglessi-Markopoulou O (2001). One-pot synthesis of optically active tetramic acids from amino acids mediated by 1-hydroxybenzotriazole.

[23] Booth PM, Fox CMJ, Ley SV (1987). Preparation of acyltetronic acids using t-butyl acetothioacetate: total synthesis of the fungal metabolites carolic, carlosic, and carlic acids.

[24] Jones RCF, Duller KAM, Vulto SIE (1998). 1,3-Dipolar cycloaddition route to oxygen heterocyclic triones.

[25] Mittra A, Yamashita M, Kawasaki I, Murai H, Yoshioka T, Ohta S (1997). A useful oxidation procedure for the preparation of 3-alkanoyltetronic acids.

[26] Schobert R, Muller S, Bestmann H-J (1995). One-pot synthesis of *α*, *γ*-disubstituted tetronic acids from *α*-hydroxyallyl esters: a novel “tandem-wittig-claisen”-reaction.

[27] Effenberger F, Syed J (1998). Stereoselective synthesis of biologically active tetronic acids.

[28] Buhler H, Bayer A, Effenberger F (2000). A convenient synthesis of optically active 5,5-disubstituted 4-amino- and 4-hydroxy-2(5H)-furanones from (S)-ketone cyanohydrins.

[29] Mitsos CA, Zografos AL, Igglessi-Markopoulou O (2000). Regioselective ring opening of malic acid anhydrides by carbon nucleophiles. Application in the synthesis of chiral tetronic acids.

[30] Schobert R, Gordon GJ, Bieser A, Milius W (2003). 3-Functionalized tetronic acids from domino rearrangement/cyclization/ring-opening reactions of allyl tetronates.

[31] Mallinger A, Gall TL, Mioskowski C (2009). 3-Aryltetronic acids: efficient preparation and use as precursors for vulpinic acids.

[32] Athanasellis G, Igglessi-Markopoulou O, Markopoulos J (2002). Novel short-step synthesis of optically active tetronic acids from chiral *α*-hydroxy acids mediated by 1-hydroxybenzotriazole.

[33] Kaufmann GF, Sartorio R, Lee SH (2006). Antibody interference with N-Acyl homoserine lactone-mediated bacterial quorum sensing.

[34] Kaufmann GF, Sartorio R, Lee S-H (2005). Revisiting quorum sensing: discovery of additional chemical and biological functions for 3-oxo-N-acylhomoserine lactones.

[35] Lowery CA, Park J, Gloeckner C (2009). Defining the mode of action of tetramic acid antibacterials derived from Pseudomonas aeruginosa quorum sensing signals.

[36] Yendapally R, Hurdle JG, Carson EI, Lee RB, Lee RE (2008). N-substituted 3-acetyltetramic acid derivatives as antibacterial agents.

[37] Hurdle JG, Yendapally R, Sun D, Lee RE (2009). Evaluation of analogs of reutericyclin as prospective candidates for treatment of staphylococcal skin infections.

[38] Gallagher RT, Richard JL, Stahr HM, Cole RJ (1978). Cyclopiazonic acid production by aflatoxigenic and non-aflatoxigenic strains of *Aspergillus flavus*.

[39] Steyn PS, Rabie CJ (1976). Characterization of magnesium and calcium tenuazonate from Phoma sorghina.

[40] Lebrun M-H, Duvert P, Gaudemer F (1985). Complexation of the fungal metabolite tenuazonic acid with copper (II), iron (III), nickel (II), and magnesium (II) ions.

[41] Fujita M, Nakao Y, Matsunaga S (2001). Ancorinosides B-D, inhibitors of membrane type 1 matrix metalloproteinase (MT1-MMP), from the marine sponge Penares sollasi Thiele.

[42] Biersack B, Diestel R, Jagusch C, Sasse F, Schobert R (2009). Metal complexes of natural melophlins and their cytotoxic and antibiotic activities.

[43] Laursen M, Bublitz M, Moncoq K (2009). Cyclopiazonic acid is complexed to a divalent metal ion when bound to the sarcoplasmic reticulum Ca^2+^-ATPase.

[44] Markopoulou O, Markopoulous J, Nicholls D (1990). Synthesis of 3-butanoyl- and 3-benzoyl-4-hydroxy-3- pyrrolin-2-ones and their complexes with metal ions.

[45] Trzeciak AM, Ziolkowski JJ (1994). 1,5-Hexadiene selective hydroformylation reaction catalyzed with Rh(acac){P((OPh)_3_}_2_/P(OPh)_3_ and Rh(acac)(CO)(PPh_3_) / PPh_3_ complexes.

[46] Heaton BT, Jacob C, Markopoulos J (1996). Rhodium(I) complexes containing the enolate of N-acetyl-3-butanoyltetramic acid (Habta) and the crystal structure of [Rh(abta){P((OPh)_3_}_2_].

[47] Petroliagi M, Igglessi-Markopoulou O, Markopoulos J (2000). Complexation and spectroscopic studies of 5-Benzylidene-3-hexanoyl tetramic acid (BHTA) with magnesium (II), zinc (II) and barium (II) ions.

[48] Imamura N, Adachi K, Sano H (1994). Magnesidin A, a component of marine antibiotic magnesidin, produced by Vibrio gazogenes ATCC29988.

[49] Gavrielatos E, Mitsos C, Athanasellis G (2001). Copper(II), cobalt(II), nickel(II) and zinc(II) complexes containing the enolate of N-acetyl-3-butanoyltetramic acid (Habta) and its phenylhydrazone derivative analogues. Crystal structure of [Cu(abta)_2_(py)_2_] · 2H_2_O.

[50] Keiichi M, Sakie H, Masato M, Satoru H

[51] Sakie H, Keiichi M, Masato M, Satoru H

[52] Lippard SJ, Berg JM (1994).

[53] Hadjiliadis N, Sletten E (2009).

[54] Gavrielatos E, Athanasellis G, Igglessi-Markopoulou O, Markopoulos J (2003). Cationic diamineplatinum(II) complexes containing the enolate of N,3-acetyl-4-hydroxypyrrolin-2-one.

[55] Gavrielatos E, Athanasellis G, Heaton BT (2003). Palladium(II)/*β*-diketonate complexes containing the enolates of N-acetyl-3-acyltetramic acids: crystal structure of the Lewis base adduct, [Pd(py)_4_](abta)_2_.

[56] Tanaka K, Matsuo K, Nakaizumi Y (1979). Structure-activity relationships in tetronic acids and their copper(II) complexes.

[57] Kawai H, Imaoka T, Hata G Process for the production of antitumor platinum complexes.

[58] Lusty JR, Pollet P (1983). Palladium complexes involving tetronic acid derivatives.

[59] Manku GS, Gupta RD, Bhat AN, Jain BD (1970). Physicochemical investigations of some bivalent ion complexes with oximidobenzotetronic acid and their comparison with the corresponding 2-nitroso-1-naphthol complexes.

[60] Reck G, Schultz B, Zschunke A, Tietze O, Haferkorn J (1994). Crystal structures of nickel(II) and copper(II)-Schiff-bases complexes with tetramic and tetronic acid subunits.

[61] Markopoulos J, Athanasellis G, Zahariou G, Kikionis S, Igglessi-Markopoulou O (2008). Coordination behavior of 3-Ethoxycarbonyltetronic acid towards Cu(II) and Co(II) metal ions.

